# A Phenotypic Mouse Model of Basaloid Breast Tumors

**DOI:** 10.1371/journal.pone.0030979

**Published:** 2012-02-09

**Authors:** Soyoung Kim, Avtar Roopra, Caroline M. Alexander

**Affiliations:** 1 McArdle Laboratory for Cancer Research, University of Wisconsin-Madison, Madison, Wisconsin, United States of America; 2 Department of Neuroscience, University of Wisconsin-Madison, Madison, Wisconsin, United States of America; Sanford Burnham Medical Research Institute, United States of America

## Abstract

Chemotherapeutic strategies that target basal-like breast tumors are difficult to design without understanding their cellular and molecular basis. Here, we induce tumors in mice by carcinogen administration, creating a phenocopy of tumors with the diagnostic and functional aspects of human triple negative disease (including EGFR expression/lack of erbB, estrogen-independent growth and co-clustering of the transcriptome with other basaloid models). These tumor strains are a complement to established mouse models that are based on mutations in Brca1 and/or p53. Tumors comprise two distinct cell subpopulations, basal and luminal epithelial cells. These cell fractions were purified by flow cytometry, and only basal cell fractions found to have tumor initiating activity (cancer stem cells). The phenotype of serially regenerated tumors was stable, and irrespective of tumor precursor cell. Tumors were passaged entirely *in vivo* and serial generations tested for their phenotypic stability. The relative chemo-sensitivity of basal and luminal cells were evaluated. Upon treatment with anthracycline, tumors were effectively de-bulked, but recurred; this correlated with maintenance of a high rate of basal cell division throughout the treatment period. Thus, these tumors grow as robust cell mixtures of basal bipotential tumor initiating cells alongside a luminal majority, and the cellular response to drug administration is dominated by the distinct biology of the two cell types. Given the ability to separate basal and luminal cells, and the discovery potential of this approach, we propose that this mouse model could be a convenient one for preclinical studies.

## Introduction

After subtracting ER+/PR+ and erbB2+ breast tumors, there remains a miscellaneous collection of so-called “triple negative” tumors (15–20% of total tumors) that are ill defined with respect to their molecular and cellular basis. The majority of these triple negative tumors are also basaloid [1], [2], [3], [4], [5], [6], as defined by their relative over-representation of lineage-specific markers from the basal mammary epithelial cell lineage, including cytokeratin 5 (KRT5) and EGFR. When analyzed, immunocytochemical localization of cognate basal proteins shows that expression of basaloid markers is heterogeneous, and restricted to a sub-population of the total tumor cell population [6], [7]. Luminal-associated markers are also heterogeneously expressed, and overall, the tumors therefore comprise mixtures of basal-like and luminal-like cells [8].

Other breast tumor types have known molecular etiologies offering effective therapeutic targets. The hunt is on for similar targets in this tumor type, often by searching for consistent genetic changes, either at the level of DNA mutations or transcriptional signatures [2], [9], [10], [11], [12]. Studies have focused on the exclusively triple negative breast tumors that arise in women with familial Brca1 mutations. Mutations in Brca genes occur in approximately 1/5 triple negative tumors [13], and this pathway appears to be affected in other ways (“BRCA-ness” [2], [10]) to create deficiencies in DNA repair (and opportunities for synthetic lethal drug development). As a marker of prevalence for this common etiology, a signature associated with functional assays have shown that homologous recombination is deficient in 2/3 triple negative tumors [14], and an analysis of the prevalence of a DNA repair signature has derived an approximately similar estimate [15].

With good reason, several groups have focused on the clear association of Brca1 and p53 mutations with triple negative human breast tumors to build models in mice [16]. However, it is not likely that these mutations will cover all the molecular drivers for this disease. To generate alternatives, we chose instead to focus on recapitulating the dominant phenotype of mixed basal-luminal cell populations, and to use the random mutational screen afforded by carcinogen administration to select the tumor driver that induces this phenotype. This offers a couple of advantages, one is that carcinogen administration may provide a relevant etiology for breast cancer, and the second is that random mutation offers the opportunity for tumor driver discovery in the future.

Since basal and luminal cells are the usual progeny of basal-associated stem cells [17], [18], it was quickly assumed that these tumors develop as the result of disordered stem cell growth and differentiation. Whilst the importance of tumor stem cells to the growth and metastasis of breast tumors *in situ* is not completely understood, it is clear that these cells have different cellular properties from the majority tumor. Thus, drug treatments that do not substantially affect tumor growth can severely deplete the tumor initiating cell population [19], [20]. Other studies have shown that cell minorities that are adapted to survive genotoxic, metabolic or hypoxic stress (either with or without radiation or chemotherapeutic treatment) may be important to tumor recurrence [21], [22], [23]. A clear take home message from studies of heterogeneous tumor cell populations is the importance of understanding the separate and individual biology of the constituent cell populations, to give insight into drug resistance and tumor recurrence.

By choosing a phenotypic model of basal-luminal cell mixtures, we assume that these mixtures are in some way important to the tumor growth and development, rather than co-existing because of a passive random process of differentiation. The model we have developed for this purpose relies upon a classical rodent model of breast cancer that has been used for several decades to test potential breast tumor chemotherapeutics or preventative strategies [24]. Thus, tumors induced in rodents by the polycyclic aromatic hydrocarbon, 7,12-dimethylbenz(α)anthracene, (DMBA) are fast-growing and contain mixtures of basal and luminal-like cells. Here, we show that these “DTumors” (DMBA-induced tumors) are functionally triple-negative, and therefore do not depend upon the two pathways that are the known drivers of human breast tumor growth. These tumors can be serially passaged using low numbers of cells as tumor initiating populations, and re-grow rapidly and consistently. The constituent cell types can be separated by flow cytometry and individually assessed for their functionality with respect to tumor propagation. In contrast to the Brca1/p53 models described above, the cancer stem cells are restricted to the basal cell fraction, their histology and transcriptome are different, and tumor cells assemble Rad51-associated foci in response to activation of DNA damage repair. Furthermore, we show that the response of both cell types to anthracycline administration is unequal, that the growth rate of the basal minority is not affected by chemotherapeutics, and tumors recur.

## Results

### Carcinogen-induced mammary tumors share the principal features of human-basal-like breast tumors

The principal features of basaloid tumors are 1) over-expression of mRNAs typically expressed by basal cells and 2) triple negative character (ERα, PR-negative; erbB2 negative). We therefore examined these criteria at the molecular and functional levels.

**Figure 1:**
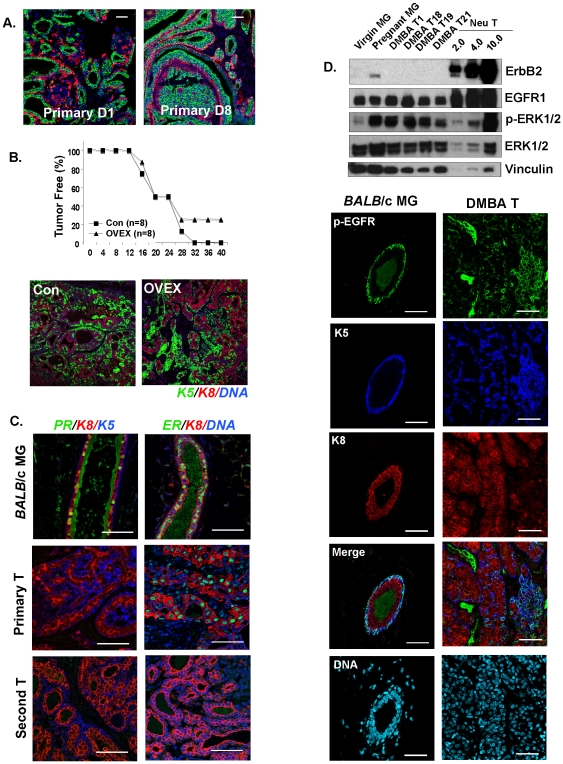
KRT5+ DTumors parallel human basal-like breast tumors by functional and molecular assay. (A) *Mixed lineage tumors*. 19 primary tumors were double stained with lineage-specific markers (keratin 5, basal cells, stained red; keratin 8, luminal cells, stained green) and 17 primary tumors (90%) comprise ≥10% K5-positive cells, as well as K8-positive cells, indicating that these tumors are bi-lineal. Two representative images from two different primary tumors are shown. Scale bar = 50 µm. (B) *Estrogen-independence.* The appearance of palpable tumor masses was measured after transplanting 10,000 tumor cells into either control or ovarectomized three-week old BALB/c recipients (representative of 3 strains of primary tumor). Re-growth of tumors is no different in recipients grafted at 3 weeks of age. Paraffin sections from tumors removed from ovariectomized hosts were compared to tumors from normal hosts, by double staining for K5 (green) and K8 (red). There was no difference in their basal/luminal constituent cell types. (C) *Experimental tumors are ERα/PR-negative*. Paraffin sections from normal virgin BALB/c mammary glands (MG), together with an example of a primary tumor (Primary T) and a secondary tumor (Second T), were stained for ERα and PR, and counterstained as indicated with either K5 (basal) or a DAPI nuclear stain. (The specificity of the anti-PR-A staining procedure is illustrated; Fig. S7). (D) *EGF signaling receptor expression.* To evaluate expression of erbB2 and EFGR1, together with a downstream effector of EGFR1, p-ERK1/2 (and total ERK1/2) in four primary tumors (DMBA D1, D18, D19, and D21), tumor tissue lysates (20 µgs) were compared with tumor tissue lysates from erbB2/neu transgenic mice (with 10.0, 4.0 and 2.0 µgs total protein), and with mammary gland from mid-pregnant and virgin mice. Vinculin was used as a loading control. Immunohistochemical staining for pEGFR in paraffin sections from normal mammary glands and tumors confirmed and extended the Western blotting results. Cell surface-associated pEGFR is typical of basal cells in normal mammary glands and this cell type-specific expression pattern is conserved in basaloid tumor cells. (Note that the green stain in the lumens is an artifact associated with sticky luminal secretions; panels C and D).

It has been shown before that DMBA-induced mouse tumors comprise mixtures of basal and luminal-like cells [30]. We have confirmed this using keratin expression (most often for this study, cytokeratin 5 marking basal cells (KRT5) and cytokeratin 8 for luminal cells; Fig. 1A). Typically, mouse models are not considered to be good models of estrogen-dependent tumors (except those that over-express ERα [31], and one strain bearing a mutant p53 allele [32]), mainly because, in contrast to human tumors, these mouse tumors are estrogen-independent despite the presence of ERα-positive cells [33]. To test whether DTumors depend upon the estrogen-ERα growth axis, we therefore applied a functional standard. 10 k tumor cells were transferred into fat pads of either ovarectomized or normal recipient mice (Fig. 1B). This analysis showed that tumor growth was not stimulated by estrogen, and that the basal/luminal cell mixtures typical of these tumors were estrogen-independent. (We attributed the failure to grow of 20% of grafts to technical issues). Stains for ERα, and the canonical target gene, PR, showed that although primary tumors were scored as ERα-positive, there was little expression of PR (Fig. 1C). Grafts of cells from primary tumors grew out slowly, and by the time of sampling, the secondary (and subsequent generations of) tumors were entirely ERα-negative (Fig. 1C). The elimination of residual ERα expression during serial outgrowth suggests negative selection, and indeed tumors grow faster in estrogen-low environments than in normal mice (data not shown).

The last criterion of the triple negative description is that tumors be erbB2-negative/EGFR-positive [2], [3]. To test whether this was true, tumor lysates were analyzed by Western blotting for their relative expression of erbB2 (not detectable) and EGFR. EGFR protein, and an activated downstream effector, pERK1/2, were expressed at levels typical of normal mid-pregnant mammary epithelium, where it is known to be necessary and sufficient for growth induction [34] (Fig. 1D). These lysates were compared with lysates from Neu/erbB2/HER1-induced mouse mammary tumors, where the erbB2 signaling pathway is the tumor driver. The level of EGFR expressed by DTumors was less than expressed by Neu-tumors (approximately 100× less per µg total protein), but the activation of p-ERK was much higher for (approximately) corresponding receptor levels. This may relate to recent data that shows a requirement for EGFR for other signaling events, such as FGFR-mediated oncogenesis (also resulting in ERK activation) [35]. By immunohistochemistry, activated phospho-EGFR co-localizes with basal cells (shown by double staining with KRT5), suggesting that these cells have more active, constitutive EGFR signaling (Fig. 1D). This is consistent with recent studies showing that EGFR signaling is required to induce and maintain normal basal/myoepithelial mammary epithelial cells [36], and activated in basaloid human tumors and cell lines [37]. For human basaloid tumors, EGFR is typically expressed by subpopulations of tumor cells (where it is also membrane associated), as summarized by Badve et al. [2].

**Figure 2:**
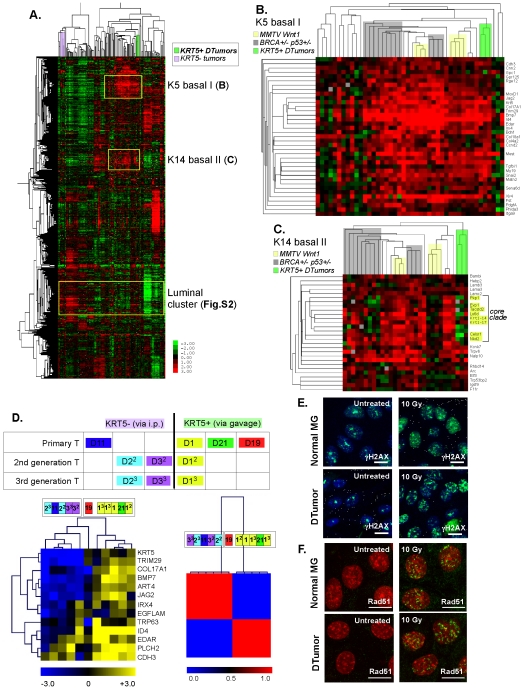
DMBA-induced KRT5+ mammary tumors express a basal signature. (A). To test the relatedness of tumors by transcriptional profiling, we compared samples of DTumors representing the two tumor classes (the specifics of these samples are shown in (D), *KRT5+ DTumors* and *KRT5- DTumors*) with a group of 122 reference tumors described by Herschkowitz et al [30]. Excerpted here are the two key basaloid signatures, keratin5 basal I signature (B) and keratin14 basal II signature (C). The three principal groups of mouse basaloid tumors known to date (MMTV-Wnt1, Brca1/p53 and these DTumors) are indicated in different colors, forming clades that are related by their basal I K5 signature and differentiated by their basal II K14 signature. For comparison, another group of DMBA-induced tumors induced in BALB/c mice by intraperitoneal injection (five *KRT5- DTumors*; luminal tumors that are KRT5-negative, see Methods) co-clustered with luminal tumors developing in transgenic strains (Fig. S2). D) *Robust serial regeneration*. To compare these two DTumor groups with each other, these 10 tumors (five KRT5-positive basaloid tumors and five KRT5-negative tumors) were compared using the essential basal I signature (comprising 13 genes; LHS panel; Herschkowitz et al [30]), together with an unbiased consensus clustering analysis (RHS). Primary tumors were numbered (D1, D2 etc), and samples of each were taken at different passage numbers labeled as superscript (secondary D1^2^ and tertiary D1^3^ etc), to test the relatedness between samples and between generations. E–F) *Assay of Brca1-dependent initiation of homologous recombination DNA repair*. Cultured cells from either normal mammary gland or a DTumor were cultured for 2 days, and irradiated with 10 Gy, 6 hours prior to imunohistochemical staining for γH2AX (green) and DNA (blue) (E; to illustrate the cellular recognition of DNA damage), and the activation of homologous recombination-mediated DNA repair (F; assembly of Rad51-associated foci, Rad51, green; DNA, red). Scale bar = 10 µm.

For BALB/c mice administered DMBA by gavage, most tumors have a remarkably consistent appearance by histological criteria (Fig. 1A; out of 19 analyzed, 17 were KRT5-positive), and are described as adenocarcinomas with acinar differentiation [38]. To test their phenotype by genetic means, we evaluated their characteristics and relatedness by transcriptional profiling. To do this, we compared DTumors (induced by orogastric gavage; KRT5-positive) with tumors induced by intraperitoneal injection of DMBA into BALB/c mice (see Methods; typically undifferentiated adenocarcinomas; KRT5-negative; Fig. S1). This cohort therefore arises in the same strain, using the same carcinogen, to provide a contrasting phenotype in the same strain background. Samples of primary tumors, together with tumor strains at different generation numbers, were submitted to the Perou lab for analysis, as per Herschkowitz et al [30]. When both tumor types were co-clustered with a reference library of 122 tumor samples, arising in various mouse breast tumor models [30], samples designated as KRT5+ clustered together with other basaloid tumor models (Fig. 2A–C), and those designated as KRT5- clustered together with luminal models, such as transgenic MMTV-*neu*-induced tumors (Fig. 2A and Fig. S2).

There are two principal signatures associated with basaloid tumors, basal I (containing KRT5) and basal II (containing KRT14) signatures. Heat maps of these two key signatures are shown in Figs. 2A–C. The expression of the KRT5 signature was consistent across the basaloid mouse models analyzed (Brca1/p53 and Wnt1, alongside DMBA-induced tumors in FVB mice and DTumors induced in BALB/c model, this study, Fig. 2B). Interestingly, the core KRT14 signature divided the Brca1/p53 and Wnt cohorts into two types, and suggested that the DTumors fall into the KRT14-low subtype (Fig. 2C).

To compare the relationships between the 10 BALB/c tumor samples analyzed here, the KRT5 basal I signature was used to conduct an unsupervised hierarchical clustering of five KRT5-positive DTumors and five KRT5-negative tumors (Fig. 2D). The diagnostic genes for this group (which include collagen17, BMP7, Jagged2, Id4, ectodysplasin A, EGFLAM (an ECM component), Iroquois homeobox4, P-cadherin and phospholipaseC ε2) resolved the 10 tumors into the KRT5-positive and negative groups. Consensus clustering (using the same gene set) generated 2 sample groups that also broadly recognized the KRT5-positive and -negative tumors; the exception was the KRT5-positive tumor (D19) that was intermediate between the two groups by all statistical assays.

Since the unsupervised clustering analysis separated Brca1/p53 tumors from DTumors, we asked whether the gene set that distinguished Brca/p53 tumors from the rest of the group of 122 (excluding the DTumor series) was expressed by DTumors, and found that it was not (data not shown). The histopathology of DTumors is also dissimilar from Brca1/p53 mutant mouse tumors. They do not share the high degree of nuclear polymorphism, mitotic index (sometimes 80% of tumor cells), inflammatory infiltrate, central necrosis and pushing margins that characterize Brca1/p53 tumors (described in detail in Fig. S3). Since histopathology is usually related to molecular etiology, this data is also consistent with a different molecular basis for DTumors.

Loss of Brca1/p53 function results in a specific lesion in homologous recombination (HR), one of the pathways activated in response to DNA damage. Molecular analysis has shown that Brca1 is required to assemble DNA repair-associated nuclear foci, containing Rad51 [39]. In fact, the lack of assembly of Rad51-positive nuclear foci can be used to test the status of HR-mediated DNA damage repair [14]. To test directly whether DTumors are deficient for homologous recombination, cells from DTumors were cultured for 2 days, exposed to 10 Gy irradiation, and tested 6 hours later for their assembly of Rad51-associated nuclear foci, as per [14]. Both normal and DTumor cells accumulated H2AX (Fig. 2E) and Rad51-associated nuclear foci (Fig. 2F), suggesting that this DNA damage pathway is likely intact. This molecular assay also supports the hypothesis that DTumors are a distinct type of basaloid tumor compared to Brca1/p53 tumors.

### Purification of basal and luminal cell-enriched populations from DMBA-induced tumors

To establish whether the heterogeneity of these tumors is the result of differentiation of a bi-potential tumor cell, rather than the recruitment of host tissues to a tumor microenvironment, we transplanted tumor cells into fat pads of ubiquitously fluorescent-GFP tagged recipients (Fig. S4), either without or with endogenous epithelium (ie. “cleared” or left intact). This experiment was designed to compare tumor cell outgrowths in the presence and absence of local endogenous epithelium, to test whether normal cells could infiltrate and significantly contribute to tumor outgrowths. By either flow cytometric analysis or immunostaining analysis, epithelial tumor cells were GFP-negative, confirming that both lineages in DMBA-induced tumors derived from tumor cells, though these tumors clearly draw on their host for the recruitment of endothelial and leukocyte accessory cells.

**Figure 3:**
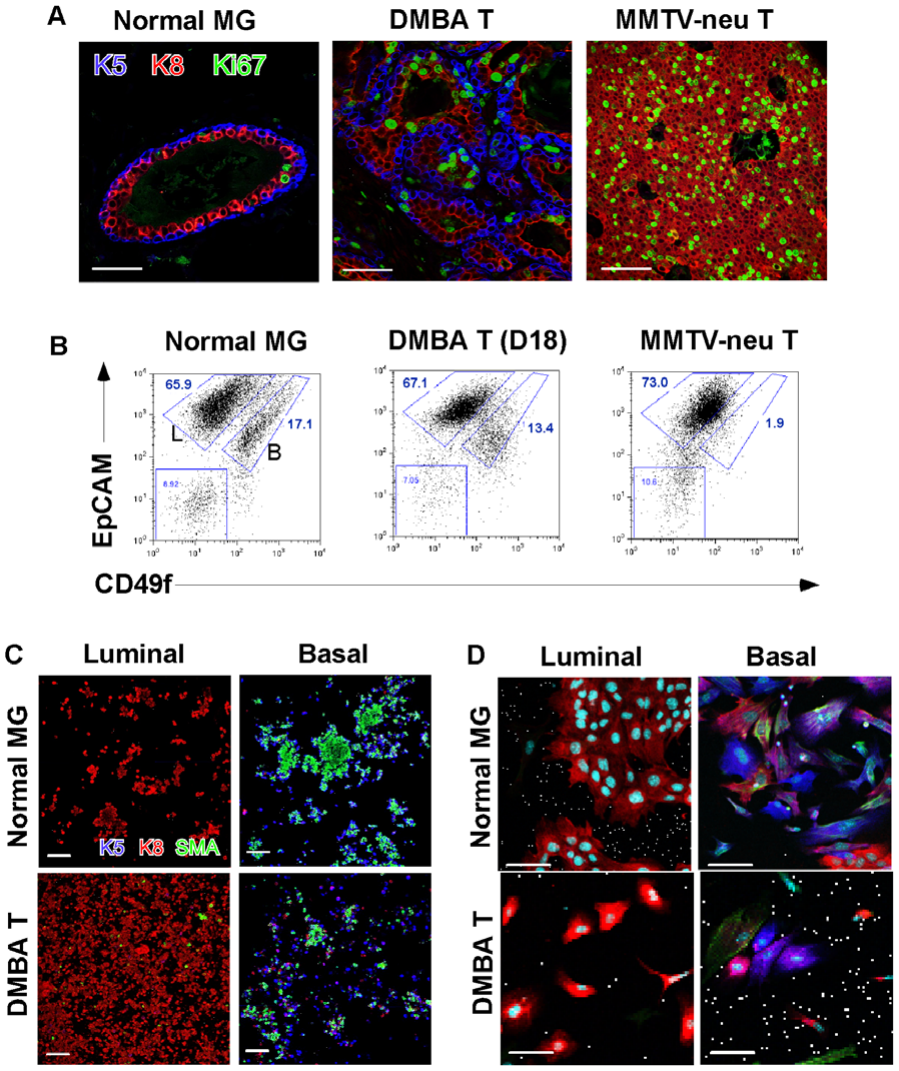
Purification of basal and luminal cell fractions from DMBA-induced mammary tumors. (A) Immunohistochemical stain of cell type specific markers (as indicated in Fig. 1), and Ki67 (mitotic marker), in the following tissues prior to dissociation; normal 12 week-old mammary glands, a DTumor, and an MMTV-*neu* induced mammary tumor. Scale bar = 50 µm. (B) Flow cytometric separation of cells from BALB/c mammary glands and DTumors using immunophenotyping antibodies, EpCAM and CD49f. Mammary epithelial cell populations from normal glands and DTumors divided into two subpopulations, an EpCAM^+^/CD49f ^low/−^ luminal (L) population, and an EpCAM^+^/CD49f ^high^ basal (B) population. The specifics of flow cytometric analysis are described in Fig. S5. (C) Immunostaining of the sorted cell fractions shown in Panel B was used to establish the relative purity of each “luminal” or “basal” cell fraction: K5 (blue), α-SMA (green), and K8 (red) (percentages are reported in the results). Scale bar = 50 µm. (D) Purified fractions of basal and luminal cells from either normal mammary gland or DTumors were cultured for 4 days and stained as for panel (C). Only basal cell fractions differentiate to both cell types (illustrating their bipotency). Compared to normal cells, tumor cells tend to co-express both keratin markers and lose their fate specification in cultures. Scale bar = 50 µm.

To separate the basal and luminal-type tumor cells, we first applied a standard method for discriminating normal basal and luminal mammary epithelial cells (cell surface expression of CD49f and CD24; [25]), but found that, despite their molecular similarities, this protocol offered little resolution. However, the cell surface antigen EpCAM, in combination with CD49f (reported originally by Lim et al [40] as a means to separate human tumor cells), provided robust discrimination (for at least 6 separate tumors, Fig. 3B and S5A; luminal cells are EpCAM^hi^CD49^lo^ and basal cells are EpCAM^lo^CD49^hi^). Though these are two distinct populations, the overlap of expression of these cell surface markers is high enough that neither alone is sufficient to discriminate the two groups of cells. Thus expression of CD49f is approximately 5× higher in basal compared to luminal cells, and EpCAM is 7× higher in luminal compared to basal cells. MMTV-*neu* tumors do not contain a similar basal cell population, either by immunohistochemistry (Fig. 3A) or flow cytometry (Fig. 3B).

When each population was tested (by “cytosplat”) for expression of luminal and basal markers in basal and luminal fractions respectively, the purification was >99% for normal populations, and >99% for the luminal cell component of tumors, dropping to 90–95% for the basal cell compartment (Fig. 3C). The basal cell component of primary tumors varied from 5–30% of the total epithelial cell population.

To evaluate the differentiation potential of each population, the luminal and basal fractions from normal and tumor cell populations were transferred to culture, allowed to grow for 4 days and stained for their expression of lineage markers. Normal luminal cells in these culture conditions do not “retro”-differentiate to express basal cell markers (Fig. 3D), whereas the bipotency of the basal cell fraction is associated with the appearance of more than 30% of cells expressing luminal cell-associated markers (after 4 days in culture). When DTumor cells were transferred to culture, a similar pattern was observed, where the luminal cells did not express basal markers, but basal cells (though only 90% pure) expressed luminal cell markers. In other words, these results are consistent with the suggestion that the potential for genomic plasticity is not significantly affected by the tumorigenic phenotype.

### Functional tumor-initiating cells co-purify with the basal tumor epithelial cell population

To directly address whether either cell population could initiate tumor growth after isografting, cell fractions purified by flow cytometry were transplanted into cleared fat pads at limiting dilutions (Table 1). Total cell populations were compared to luminal and basal cell subpopulations. All the tumor initiating cell activity was associated with the basal cell fraction population (and could be retrieved quantitatively from that fraction). Thus, for 26 recipient mice transferred with various numbers of basal cells (totaling 10 520 cells), there were 9 tumor takes. For 24 recipient mice transferred with luminal cells (totaling 23 400 cells), there were no tumor takes.

**Table 1:** Functional analysis of cancer stem cell activity in DMBA-induced mammary tumors.

Tumors	Cell fraction	% cells in fraction	No. cellstransferred	Takerate	Frequency of TICs(95% CI)
**Primary tumor** **D19**	**Total** **(Lin-)**	100	3200	2/4	1/4938(1/1574–1/15488)
			1600	1/4	
	**Luminal**	56.4	1600	0/4	zero
			800	0/4	
	**Basal**	16.2	1600	3/4	1/1593(1/589–1/4309)
			800	1/4	
**Second generation tumor D19^2^**	**Luminal**	64	1300	0/4	zero
			650	0/4	
	**Basal**	12.2	120	1/4	1/278(1/67–1/1143)
			40	1/4	
**Second** **generation tumor D1^2^**	**Luminal**	70.2	1000	0/4	zero
			600	0/4	
	**Basal**	7.0	100	1/2	1/70(1/19–1/249)
			10	2/8	

The frequency of tumor initiating cells (TICs) was compared for the total cell population (designated Lin^−^), and two cellular sub-fractions (luminal and basal cell populations), using fat pad assay of limiting dilutions of cell suspensions. Three series of cell populations were used: **Primary tumor-D19** (Fig. 2), a **second generation tumor-D19^2^** (obtained from basal cell transplantation of primary tumor D19), and another **second generation tumor-D1^2^** derived from transplantation of a different primary tumor, D1 (Fig. 2).

**Figure 4:**
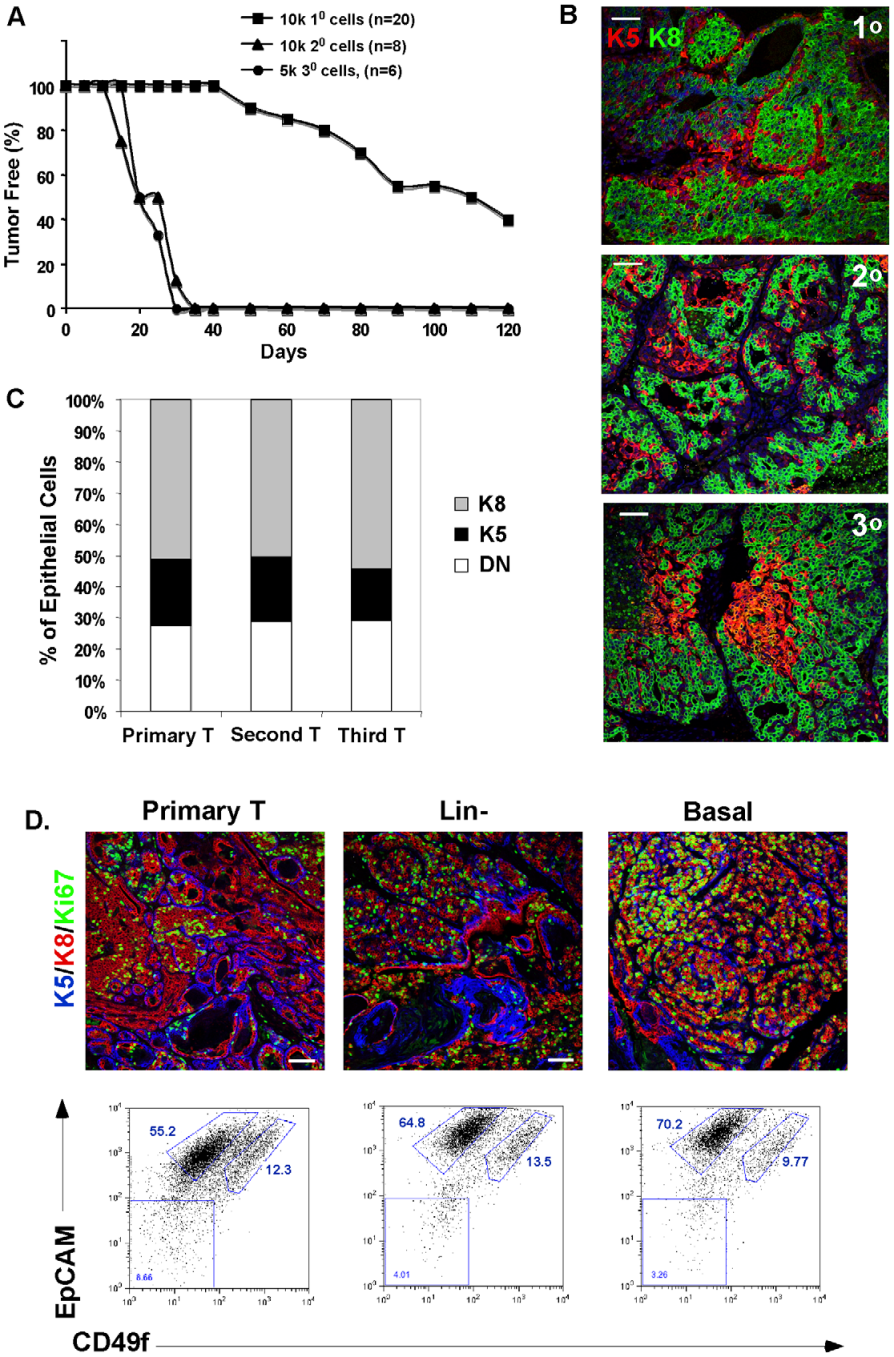
The tumor bi-lineal phenotype is stable over several generations and independent of tumor precursor cell. (A) Tumor re-growth in serial transplantation was assayed over time, by palpation (generating a Kaplan-Meier curve; n = sample number), inoculating with the cell numbers indicated. (B) Immunostaining of primary, secondary, and tertiary DTumors with anti-K5 (red) and anti-K8 (green) antisera. Scale bar = 50 µm. (C) The proportion of cells staining for each lineage marker *in vivo* (assayed from 3 separate tumors for each generation; 3 fields each) for each of the primary tumor and two serially regenerated tumors (Second T and Third T). (D) Corresponding immunocytochemical analysis and flow cytometric analysis of tumor cell populations from a primary tumor, for comparison with tumors regenerated from cell isografts of limiting dilutions of the cell type indicated (Lin^−^ and basal cell). Scale bar = 50 µm.

After flow cytometric analysis, the overall tumor initiating cell (TIC) frequency was approx 1 in 5000 cells (for the primary tumors). In secondary tumors (that are ERα-negative (Fig. 1C) and fast-growing (Fig. 4A)) the frequency of TICs increases, but the tumor initiating activity remains focused entirely in the basal fraction. For the two samples shown here, the activity rises from 1 in 1600 basal cells, to approx 1 in 70–300 basal cells (increasing 5–20×).

### The tumor phenotype is stable over several generations

The evaluation of the efficacy of chemotherapeutics for the prevention and treatment of primary carcinogen-induced tumors has previously been limited by the long latency of tumor development in response to DMBA (>200 days; [27]), and the inefficiency of a model with a low penetrance phenotype. However, we have exploited the fact that the primary tumors grow quickly after they develop. Thus, we tested how many cells were required to get rapid and consistent tumor development, and whether the tumor phenotype changed with serial regeneration.

Primary tumors were dissociated, and 10 k cells were re-inoculated into cleared fat pads. When tumors re-grew, the passage was repeated to generate a sequential series (Fig. 4A–C). The first passage of tumor cells showed considerable mouse-mouse heterogeneity in the rate of re-growth (onset 50 days, up to 4 months and beyond). However, subsequent passages grew back at a rapid and consistent pace, starting in only 20–30 days, with 100% take rate for 10 k cells. The tumors that grew up showed highly reproducible proportions of each cell type (ratios varied from 4–8:1 luminal: basal cells), both from mouse to mouse (data not shown) and from generation to generation (Fig. 4B and C; also reflected in the microarray data shown in Fig. 2).

We compared whether tumors that re-grew from limiting dilutions of purified subpopulations were different from those that re-grew from total (Lin-) mixed cell populations. The source of tumor precursor cells had no noticeable effect on tumor phenotype, and re-established the original diversity of luminal and basal cells, as measured by flow cytometry and keratin-immunophenotyping (Fig. 4D). We deduce that even after serial regeneration from limiting dilutions of cancer stem cells (presumably clonal), the re-growth activity remains focused in the basal cell fraction, and these cancer stem cells recreate a characteristic and robust diversity of mammary epithelial cells.

### The basal-like cell fraction is relatively chemo-resistant

**Figure 5:**
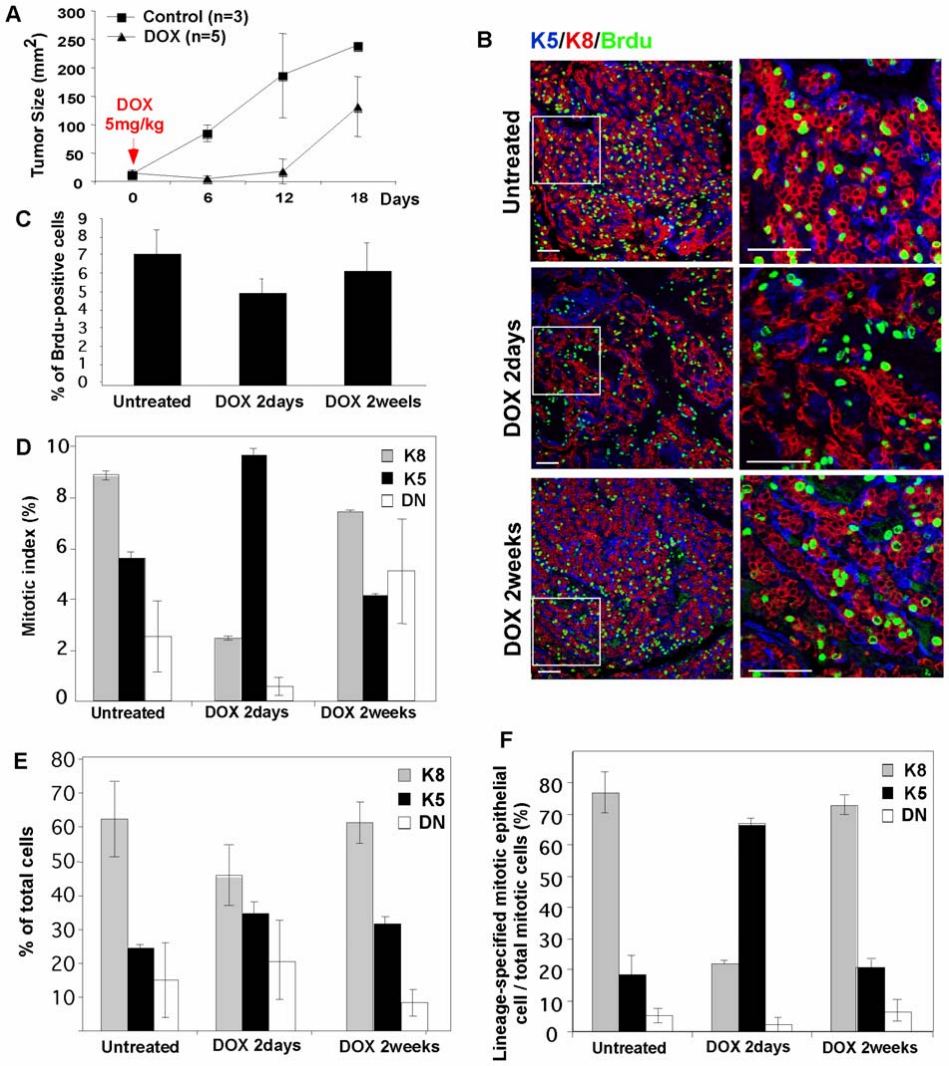
The basal cell fraction is relatively resistant to chemotherapeutics. (A) Tumor response to treatment with doxorubicin. Dissociated, second generation tumor cells (D19 shown here; results representative of 3 strains; 10 k cell inoculae) were iso-grafted into cleared fat pads, and the re-growth of tumors was observed by palpation and caliper measurement. Doxorubicin (single dose, 5 mgs/kg) was administered when tumors were 5 mm diameter (indicated as day 0), and this cohort compared to controls. Though initially substantially responsive to chemotherapy (2 days), after 2 weeks, tumor growth relapses. (B) *Selective arrest of luminal cells after exposure to chemotherapeutic*. The mitotic index (measured by anti-BrdU labeling, see Methods) of basal (**K5**) and luminal (**K8**) cells was measured in untreated or doxorubicin-treated tumors (at the indicated times, either 2 days or 2 weeks after administration). (C–F) The fraction of dividing cells (BrdU-positive) per total population was measured during the treatment course (C, compare to growth rate of palpable tumor mass, panel A). The mitotic index for basal and luminal cell sub-populations is measured separately (D). The proportion of each cell subtype is shown with respect to the total population (E; grey are luminal, K8-positive, and black are basal, K5-positive; double negative cells (DN) stain for neither marker). The proportion of basal and luminal cells that make up the dividing cell population during drug treatment and recovery are shown in panel (F).

To evaluate the response of this model to a chemotherapeutic currently in use to treat this subtype of tumor, mice with established tumors were administered the anthracycline, doxorubicin (Adriamycin™, a topoisomerase I inhibitor), and tumor growth measured with calipers (Fig. 5A). A single administration of doxorubicin prevented growth for 2 weeks, but subsequently, tumors recurred, with a growth rate similar to pre-treatment rates. To determine the individual cellular response, the mitotic index of basal and luminal cells were separately evaluated 2 days and 2 weeks after doxorubicin administration using BrdU labeling for 2 hours prior to tumor harvest (Fig. 5B–F).

In untreated tumors, approximately 7% of total tumor cells are dividing (Fig. 5C); when evaluated separately, the mitotic index of luminal and basal cells was approximately 8.5% and 5.5%, respectively (Fig. 5D). Since luminal cells constitute the majority of cells (approx 2/3; Fig. 5E), 80% of dividing cells in a tumor are luminal (quantified in Fig. 5F). Although the mitotic index of basal cells is lower, these cells could not be described as quiescent. (This claim is made with respect to the two most active periods of growth for normal mammary gland growth, ductal outgrowth and pregnancy, which contain 3–6% BrdU-positive cells). We emphasize this, because chemo-resistance is often ascribed to quiescent cell populations (since cytotoxic activity associated with drug administration almost always relies on cell division).

Two days after chemotherapeutic administration, the mitotic index of basal cells increased (Fig. 5B and D) and most of the dividing cells were basal (Fig. 5B and F), while the mitotic index of the K8-positive cell population was reduced by 80% (2% BrdU-positive; Fig. 5D, quantification of Fig. 5B). The relative proportions of cell types remained approximately constant (Fig. 5B and E; confirmed by flow cytometric analysis, shown in Fig. S6), and there was little enrichment for basal cells. We deduce that the basal cells continue to divide and differentiate into luminal cells to re-establish typical proportions of each cell type. After 2 weeks, the mitotic index of the luminal cell population was re-established at pre-treatment levels (Fig. 5D and F), and the tumor mass began to grow. (Note we found it difficult to demonstrate classic apoptosis or necrosis (Fig. S3 and data not shown), and conclude that there may be alternative routes of tumor remodeling, such as autophagy, that are important in this case). We conclude that basal cells are relatively resistant to this anti-mitotic chemotherapeutic, and that tumor re-growth is correlated with the lack of substantial effect of anthracyclines against this subpopulation.

## Discussion

We have shown that DMBA-induced breast tumors comprise a majority of luminal-like cells that cannot serve to propagate tumors after dissociation, together with a minority of basal cells that can act as tumor-initiating cells. This lineage hierarchy reflects the normal organization of mammary epithelial differentiation, where basal cells serve as the stem/progenitor cell type. The luminal-like cells are more sensitive to anthracycline treatment than the basal-like minority. In other words, after the administration of doxorubicin, basal cells do not stop dividing (measured as the mitotic index of KRT5-positive cells; Fig. 5B,D and F). However, since the proportion of each cell type is different, and the majority of tumor cells are luminal cells (Fig. 5E), it is not surprising that chemotherapeutics effective against luminal cells can de-bulk these basaloid tumors. However, it is also clear that without high efficacy against the regenerative basal cell subpopulation, therapeutic regimens will fail. Given our results, we propose that it may be possible to forecast durable responses for patients who have been treated with chemotherapeutics by independently scoring basal- and luminal-specific mitotic indices. (Note that K5 and K14 appear to be regulated differently in mouse and human; K5 is a stringent basal marker in mouse and assorts between basal and luminal cells in human, and the opposite may be true for K14 in human; [4], [40]).

Would this model be a significant addition to the models already available for the evaluation of triple negative breast cancer? There are a new wave of human xenograft models derived by serial transplantation in Nude mice, which recapitulate the human disease in most gross aspects, except their interaction with immune cells and the heterotypic host environment [41], [42]. There are clearly similarities and differences between the DTumor model we describe here, and other rodent models of triple negative tumors, that are summarized below. Both similarities and differences have positive associations. Similarities tend to emphasize the basaloid nature of these tumors and the relevance to the human disease; differences reveal aspects that are unique to this tumor model, that offer an alternative discovery platform.

Firstly, transcriptional profiling shows these tumors are basaloid (falling into a class alongside BRCA1 and p53 mutant mouse models), but distinct from them (with respect to a Brca1 signature). However, of the two basaloid signatures, the K14 basal II signature stratifies tumors arising in Brca1- or Wnt1- mouse models; some have a strong K14 basal II expression, the other weak. This carcinogen-induced model falls into the class with a strong K5 basal I signature, but weak K14 basal II signature. Though the significance of this is not yet known, several of the core genes are known to have functional roles in mammary tumorigenesis.

Secondly, immunocytochemical analysis shows this tumor model comprises cells that separately express either basal or luminal type cytokeratins. This is also typical of many (but maybe not all) human basaloid tumors [43], [44]. From the published data, it is not easy to determine whether the separate and distinct populations are always a feature of basaloid human tumors, since double-stained immunohistochemistry, visualizing more than one antigen for a given sample, is rarely presented. Furthermore, an antibody against “CK5/6” is often used for clinical evaluation, though it may not be the most reliable [45]. As a cell lineage diagnostic, it does not clearly discriminate basal and luminal cells. (These keratins tend not to be lineage-restricted, but more likely to indicate hyperplastic cells of either lineage). Histologically, human basaloid tumors often have pushing borders, significant lymphocytic infiltration, highly pleomorphic nuclei, medullary features, and exceptionally high mitotic indices (measured by immunocytochemical Ki67 assay) [2]. Though these features are well represented in Brca1-p53 based models [46], [47], they are not a characteristic of DTumors (Fig. S3).

Thirdly, on the point of tumor etiology, the significance of carcinogen exposure as an etiology for triple negative breast tumors is not known. Typically, the relevance of environmental exposure (for example, smoking) as an etiology for breast cancer has been controversial [48]. Though carcinogen exposure, in theory, should mutate genes at random, mammary tumors induced by DMBA gavage of BALB/c mice are remarkably homogeneous (by histological, molecular and transcriptional criteria; note that DMBA induces much more diverse tumors in FVB mice [30], [49], as does carcinogen administration (NMU) to rats [50]). This could imply a common molecular and/or cellular origin, though these are not yet known (probably not p53 [51] or Ras [52]). Our histological assay of homologous recombination (assembly of Rad51-associated foci) suggests that this DNA repair pathway is intact. However, this strain is known to be mutant for DNA protein kinase (DNA-PK) [53], [54], a key element of non-homologous end-joining (NHEJ), which may have relevance for the mutational rate and the ability to repair DNA in response to anthracycline administration. It is of some interest that it is not yet known whether the absence of Brca1 itself is the driver of tumor growth (by an unknown mechanism), or whether mutations induced by repair deficiencies are the source of the tumors. If the latter is correct, it may be difficult to find a common etiology amongst the genetic noise, and indeed the genomic studies to date have yet to reveal a dominant, connected set of genetic origins. Surprisingly, Varela et al [55] suggest that there are few genetic changes in the DNA of Brca1-negative mouse tumors that are consistent with a repair-deficiency, perhaps due to their over-expression of DNA repair gene mRNAs [15], [55], [56].

Fourthly, it has not been conclusively demonstrated that tumor stem cells are important to tumor growth *in vivo*. However, intuitively, cells that can serve to regenerate the tumor majority could become especially important after chemotherapeutic treatment. We examined which of the two purified cell populations could regenerate a tumor, and found that the tumor-initiating activity co-purified with basal cells. This contrasts with recently published examples of a luminal “plastic” cancer stem cell for Brca1/p53 mutant tumors. Thus, the earliest disorders of transcriptional phenotype that were noticed in precancerous breast from Brca1-mutant women showed up in the luminal cell population, perhaps a surprising observation given luminal cells are usually restricted to luminal fates, and these tumors have a clearly basaloid signature. However, this origin was confirmed in mouse studies, together with the co-purification of tumor stem cells with the luminal-type tumor cell [40], [47], [57]. Also in support, mammary tumors arising in luminal cells with floxed alleles of Brca1 and p53 showed cancer stem cell activity associated with a CD29^hi^CD24^med^ sub-population [57], [58], and these cells also express Nanog, a gene associated with plasticity and totipotency. Note that Pajic et al [57] discarded the hypothesis that TIC activity was drug-resistant (for Brca1/p53 mutant tumors) based on their lack of enrichment after drug treatment. To our view, enrichment is not predicted if the tumor stem/progenitor cell does not arrest, and we therefore vary in our interpretation.

“Plastic” luminal TIC activities have also been identified in mouse basaloid Wnt1- and p53-induced breast tumors [59], using CD61 as a luminal cell marker, and by our group, using the canonical Wnt receptor, Lrp5 [60]. A plastic luminal stem cell may also be inferred from the gene profiles of a Thy1^+^ tumorigenic fraction isolated from Wnt1-induced tumors [61]. Zhang et al. [62] showed that a CD29^hi^CD24^hi^ subpopulation isolated from these p53 mutant tumors resembled a luminal progenitor cell type.

Fifth, our data suggests a refinement for the approach to analyzing anthracycline resistance, namely to score chemotherapeutic responses individually for basal and luminal cells. For tumors that rely on basal cell minorities for regeneration after treatment, un-stratified response rates could be particularly misleading. Anthracyclines are commonly used to treat patients with triple negative breast tumors, and can be highly effective (for approximately 1/4 patients); however, patients with residual disease have the worst disease-free survival outcomes [63]. Genetic changes that are statistically linked to anthracycline resistance have been associated with amplifications in 8q22. Further analysis has shown that two common amplifications led to a deficiency in nuclear trafficking or the suppression of apoptosis [9], which could be reversed in cell lines *in vitro*. A basal cell-associated stress response protein, αßcrystallin, has been shown to be associated with chemo-resistance in human breast cancer patients [64]. In the carcinogen-induced basaloid tumor model reported here, the reason for the drug resistance of basal cells may be related to these mechanisms.

This carcinogen-induced model has properties that both classify it with basaloid models, and serve to distinguish it from others. The phenotype of the tumors is relatively stable over many generations, even when tumors are re-grown from limiting numbers of basal cells. This is useful from a pragmatic point of view, for providing consistency of drug response and tumor outgrowth for many mice during serial reconstitution. It is also fascinating from a theoretical point of view, since it implies a selective pressure for this combination of basal and luminal cells. When tumors recur, they have a phenotype indistinguishable from the untreated tumor; this is typical for breast tumor recurrence [65]. We hypothesize that the proportion of luminal and basal cell daughters is actively regulated and functionally important.

In conclusion, we have shown that the basal and luminal-like cells that comprise these basaloid tumors have different chemosensitivity. Given that the tumor initiating activity is entirely basal restricted for this model, we propose that understanding basal cell biology is key to effective targeting for this tumor type, and that the two lineages can be separately evaluated using simple cytokeratin markers. This basaloid breast cancer model, maintained entirely *in vivo*, could provide a valuable and complementary tool to add to the current models.

## Methods

### Ethics Statement

This study was performed in strict accordance with the recommendations in the Guide for the Care and Use of Laboratory Animals of the National Institutes of Health. The protocol was approved by the University of Wisconsin School of Medicine and Public Health Animal Care and Use Committee (Protocol Number: M01422). The University of Wisconsin's animal welfare assurance number on file with the Office for Protection from Research Risks is A3368-01. The number of mice used to perform this study was minimized, and every effort was made to reduce the chance of pain or suffering.

### Mice

The following strains of mice were used: C57BL/6-Tg(CAG-EGFP)1 Osb/J (strain 003291, expressing EGFP ubiquitously, driven by the β-actin promoter), FVB/N-Tg(MMTVneu)202Mul/J and BALB/c (Jackson Labs, Bar Harbor, ME).

### Antibodies used for Immunofluorescence and Western blotting

Methods for staining tumor tissue sections and sorted cells on slides were as described [25]. Primary antibodies used for immunofluorescence were: rabbit anti-keratin 5 (Covance, Madison, WI), rat anti-keratin 8 (Troma-I) (Developmental Studies Hybridoma Bank, University of Iowa), rabbit anti-ERα (Santa Cruz Biotechnology, Inc., Santa Cruz, CA), mouse anti-PRA (hPRα7; [26]), mouse anti-GFP, mouse anti-phospho (Y^1068^)-EGF Receptor (Cell Signaling Technology, Danvers, MA), FITC-conjugated mouse anti-α-smooth muscle actin (Sigma, St Louis, MO), mouse anti-Rad51 (Calbiochem, San Diego, CA), anti γH2AX (Upstate, Temecula, CA) and mouse anti-BrdU (Roche, Indianapolis, IN). Nuclear DNA was counterstained with TO-PRO-3 (Molecular Probes), for 10 minutes at room temperature. Immunofluorescent stains were visualized on a confocal microscope (BioRad MRC1024). The following antibodies were used in Western blotting as probes: anti-EGFR1 (Santa Cruz Biotechnology, Inc., Santa Cruz, CA), anti-erbB2 (Calbiochem, San Diego, CA), anti-p-ERK and anti-ERK (Cell Signaling Technology), and anti-vinculin (Chemicon International Inc., Temecula, CA).

### Dissociation of Normal Mammary Epithelial cells and DMBA-induced Tumor (DTumor) cells

The preparation of primary mammary epithelial cells was as described [25]. For tumor cell suspensions, the following modifications were made; tumors were finely chopped and digested for 1 hour, 37°C in supplemented Epicult-B (cat#05602 and 05603, Stem Cell Technologies, Vancouver, CA) supplemented with 5% fetal bovine serum, 300 U/ml collagenase and 100 U/ml hyaluronidase (cat#07912, Stem Cell Technologies). After lysis of the red blood cells, tumor cells were directly dissociated in 5 mg ml^−1^ Dispase II plus 0.1 mg ml^−1^ DNase I. A suspension of single cells was obtained by filtration through a 40 µm mesh.

### Induction of Tumors by Administration of DMBA, and Isograft Procedures

Carcinogen-induced primary tumors were induced by administration of 6×1 mg DMBA (DMBA was dissolved in tricaprylin at 5 mg/ml, 0.2 ml/dose, (Sigma, St. Louis, MO); approximately 0.15 µmols/gbw per treatment) by orogastric gavage to female BALB/c mice, once a week starting at 12–14 weeks. This well-established protocol was originally described by Dan Medina, Baylor College, TX, and was used in a prior study by our lab [27]. 20 mice were administered DMBA using this protocol, and 14 mice developed one or multiple mammary gland tumors within 7 months.

The tumors arising in response to a different protocol, but using the same mouse strain and carcinogen, were used as a useful cohort for direct functional and molecular comparison. Thirteen mice were administered DMBA by intraperitoneal injection at 12–14 weeks old (0.01 µmol/gbw, 10× less than the gavage protocol); 10 mice developed mammary gland tumors within 14 months. This protocol induced many types of tumors (principally lung and liver, see [27]), but more interestingly for this study, induced mammary tumors with a distinct histopathology (not microacinar, and no expression of basal cell associated cytokeratin5).

For serial transplantation experiments, tumors were dissociated, and 5000–10000 single cells were isografted into cleared fat pads as described [28]. To test the contribution of host cells to tumor outgrowth, BALB/c tumor cells were transplanted into F1 C57Bl6(EGFP): BALB/c recipients, to ensure immuno-compatibility. Tumor initiating cell frequency was calculated using limdil software (http://bioinf.wehi.edu.au/software/limdil).

### Drug administration to mice with isografted tumors

Doxorubicin (Sigma, St Louis, MO) was dissolved in saline, and when tumors were approximately 5 mm diameter after fat pad inoculation, mice were administered doxorubicin (5 mg/Kg) or vehicle, by intraperitoneal injection. To detect actively proliferating cells in tumors, 100 mg/kg BrdU was administered by intraperitoneal injection, 2 hours prior to tumor harvest.

### Flow cytometric analysis and sorting

Fluorescence-activated cells sorting (FACS) analysis was done using the FACSVantage SE (BD Biosciences, San Jose, CA) equipped with 633 nm and 488 nm lasers, and the following antibodies: APC-conjugated anti-CD45 (Cat. 559864; clone number 30-F11; 1 µg/ml), APC-conjugated rat anti-mouse CD31 (Cat. 551262; clone number MEC13.3; 1 µg/ml), FITC-conjugated CD49f (Cat. 555735; clone number GoH3; 30 µl/ml) from BD Biosciences, and PE-conjugated EpCAM (Cat. 118206; clone number G8.8; 0.5 µg/ml) from BioLegend (San Diego, CA). Propidium iodide (2 µg/ml final concentration) was added 15 minutes before cell analysis. The gating procedures are shown in Fig. S5 (details according to [29]).

### Primary cultures of DTumor cells

15,000–20,000 viable cells were seeded into Matrigel-coated 8 well-chamber slides (Nalge Nunc International, Naperville, IL), and cultured for 4 days in DMEM/F12 (Invitrogen, Carlsbad, CA) supplemented with 2% FBS (Harlan Laboratories, Indianapolis, IN), 10 µg/ml insulin (Sigma), 100 U/ml Penicillin/Streptomycin (Invitrogen), and 20 ng/ml EGF (R&D System, Minneapolis, MN). To assess DNA damage (assembly of γH2AX) and the activation of homologous recombination (Rad51 focus formation) in DTumor cells, primary cultures of cells from either normal mammary gland or DTumors were irradiated with 10 Gy, and incubated for 6 hours before fixation and immunohistochemical staining.

### Microarray analysis

Total RNA was collected and processed according to Herschkowitz et al [30]. RNA was assessed for quality as described, and analyzed on Agilent Mouse Oligo Microarrays (G4121A) by the Perou laboratory. Data was uploaded and normalized and compared to the Gene Expression Omnibus dataset listed under the series GSE3165, using the processes previously described. To examine the relatedness of the two morphologically distinct tumor types induced in *BALB*/cJ mice in response to DMBA, a set of 10 samples (five each of basaloid (KRT5-positive DTumors) and non-basaloid tumors (KRT5-negative DTumors)) were compared by consensus clustering and unsupervised hierarchical clustering Multi-Experiment Viewer (MeV_4_5 v10.2). We used a core signature of 13 basaloid genes, described by Herschkowitz et al [30]. Samples were median-centered and then normalized followed by average clustering using Euclidian distances.

## Supporting Information

Figure S1 **Different administration routes of the same carcinogen (DMBA) induce different types of tumors.** Sections of tumors arising in response to the administration of 6×1 mg DMBA by intragastric gavage (A) or by intraperitoneal injection of 70 µgs DMBA (B) were stained with H&E (top) and lineage-specific markers, keratin 5 (red) and keratin 8 (green) (bottom). The basaloid tumors (DTumors) arising in response to orogastric administration of DMBA were microacinar adenocarcinomas, staining positive for keratin-5, whereas the most frequent tumor arising in mice administered DMBA by the intraperitoneal route were keratin-5 negative and less differentiated.
(TIF)

Figure S2 **Comparison of the transcriptome of DTumors and KRT5-negative BALBc tumors with a bank of tumors from mouse models** (previously described by Herschkowitz et al [30]). The two tumor types that arise in BALB/c mice in response to DMBA are shown on the overview of the heat-map, the basaloid group (in green) and the luminal group (in purple). Details of the KRT5-positive type I basal and KRT14-positive type II basaloid signatures are outlined (and illustrated in Fig. 2A–C). Samples designated as KRT5^+^ clustered together, but one (D19) was sufficiently plastic at the transcriptional level to express both basal and luminal genes. The luminal signature, boxed as (c), was highly represented in the KRT5-negative cohort (and excluded from the basaloid tumor type) and these tumors co-cluster with luminal tumors (including MMTV-expressing models such as MMTV-neu and MMTV-PyMT). One third-generation KRT5-negative tumor had a claudin-low signature. Detailed data and label expansions are available on request.
(TIF)

Figure S3 **Histopathology of DTumors (treated with doxorubicin) and Brca1-p53 mutant mouse tumors.** H&E stained paraffin sections of a representative DTumor, either untreated, or treated with doxorubicin for 2 days, or 2 weeks (as per Fig. 5) are shown, to illustrate their microacinar substructure, relatively lower stroma/interstitium with little evidence of inflammatory cells, lower proportion of necrotic areas/cells, low nuclear pleiomorphism and mid level mitotic index (typically grade II tumors). In contrast, a representative Brca1/p53 mutant tumor (illustrated by Molyneux et al [47]) shows higher rates of necrosis, high level nuclear polymorphism, substantial inflammatory infiltrate and high mitotic indices (not shown are their pushing margins).
(TIF)

Figure S4 **Only the stromal components are recruited from host, not either of the epithelial cell subtypes.** (A) The experimental scheme to test for incorporation of cells from the host mammary gland into tumors. All cells in the host are GFP-positive, and isografted cells are unlabeled and transferred into either a cleared fat pad (no endogenous epithelium) or a normal mammary gland (with endogenous epithelium) to test whether endogenous mammary epithelial cells (say the basal cells) could be recruited to the growing tumor isograft, to generate the typical cell mixture. (B) Representative flow cytometric analysis of tumors growing in GFP-expressing recipients. Cells were stained for non-epithelial markers (so-called Lin+; CD31, (most) endothelial cells; CD45, (most) hematopoietic lineage), together with GFP (expressed by cells in the host fat pad). The quartiles represent cells that separate as follows: top right, non-epithelial tumor-associated cells derived from the host (Lin+ GFP+); bottom left, tumor epithelial cells derived from transplantation (Lin− GFP− cells). Epithelial cells recruited to the tumor from host are predicted to appear in the bottom right quartile (together with any non-epithelial cell types that turn out to be CD45−, CD31−). This assay illustrated the massive infiltration of non-epithelial GFP-positive cells to these tumors, and there was no difference between the fraction of CD31/CD45-negative cells with or without endogenous epithelium. (C) Visualization of GFP-positive cells in tumors. To confirm that GFP-positive cells were not epithelial, tumor sections were counterstained with luminal (K8, red) or basal (K5, blue) epithelial cell markers. Far left, low power; other panels are higher magnification (of area boxed); triple stained as indicated, or single stains to show cellular detail. Scale bar = 50 µm.
(TIF)

Figure S5 **Gating and description of flow cytometric analysis and sorting.** (A) More examples to display the typical separation of luminal and basal cells from several DTumors, based on their expression of EpCAM and CD49f. (B) The gating procedures used to separate luminal and basal epithelial cells from BALB/c mammary glands and DTumors. After gating out the debris and cell doublets, dead cells and lineage positive cells (Lin^+^) were sequentially excluded by staining with propidium iodide, and then CD45/CD31. The gating tree shows the proportion of events that were filtered through sequential gates. Single stained normal mammary gland epithelial cells were used as compensation controls and the automated compensation procedure were used with Diva Software. Cells were sorted with a 130 µm nozzle tip at low pressure (12 psi), and cells and 4-way sample collection tubes were maintained at 4°C.
(TIF)

Figure S6 **Flow cytometric analysis of untreated and doxorubicin-treated tumors.** A representative cytogram of the cellular constituents of tumors (untreated and 2 days after administration of doxorubicin) shows that the proportion of each cell type is robustly maintained during treatment.
(TIF)

Figure S7 **Demonstration of specificity of PRA staining.** Immunohistochemical staining for PRA in normal virgin (MG) and ovarectomized BALB/c mammary glands, counterstained as indicated with both lineage markers, K5 (basal) or K8 (luminal). PRA staining is absent in ovarectomized glands, consistent with the loss of estrogen-ERα signaling.
(TIF)
