# Supplementary figures and images for: A Phenotypic Mouse Model of Basaloid Breast Tumors
Source: PLoS One. 2012 Feb 9;7(2):e30979. doi: 10.1371/journal.pone.0030979 (PMC3276569; doi:10.1371/journal.pone.0030979)

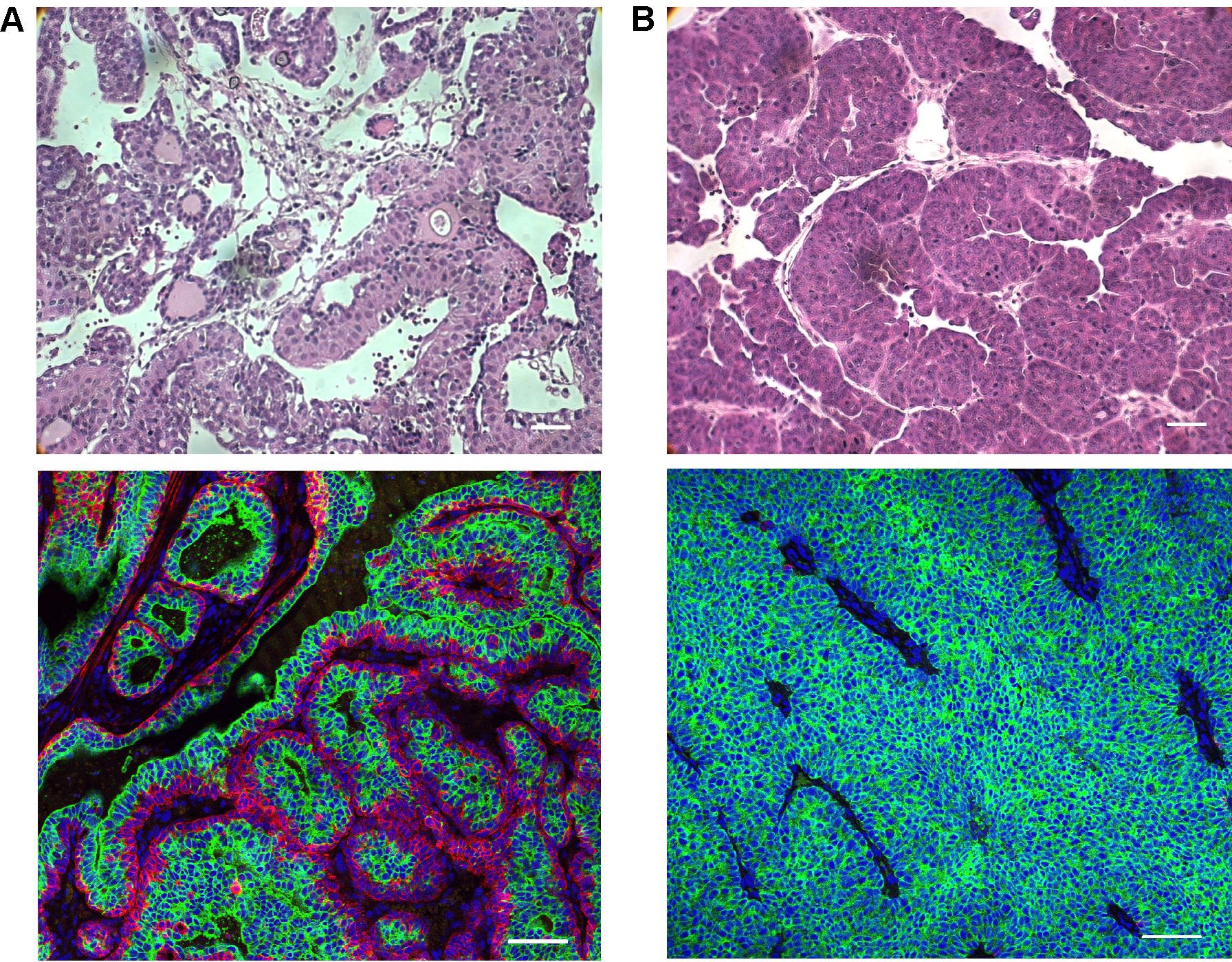

Supplement: Figure S1 — Different administration routes of the same carcinogen (DMBA) induce different types of tumors. Sections of tumors arising in response to the administration of 6×1 mg DMBA by intragastric gavage (A) or by intraperitoneal injection of 70 µgs DMBA (B) were stained with H&E (top) and lineage-specific markers, keratin 5 (red) and keratin 8 (green) (bottom). The basaloid tumors (DTumors) arising in response to orogastric administration of DMBA were microacinar adenocarcinomas, staining positive for keratin-5, whereas the most frequent tumor arising in mice administered DMBA by the intraperitoneal route were keratin-5 negative and less differentiated. (TIF) [file pone.0030979.s001.tif]

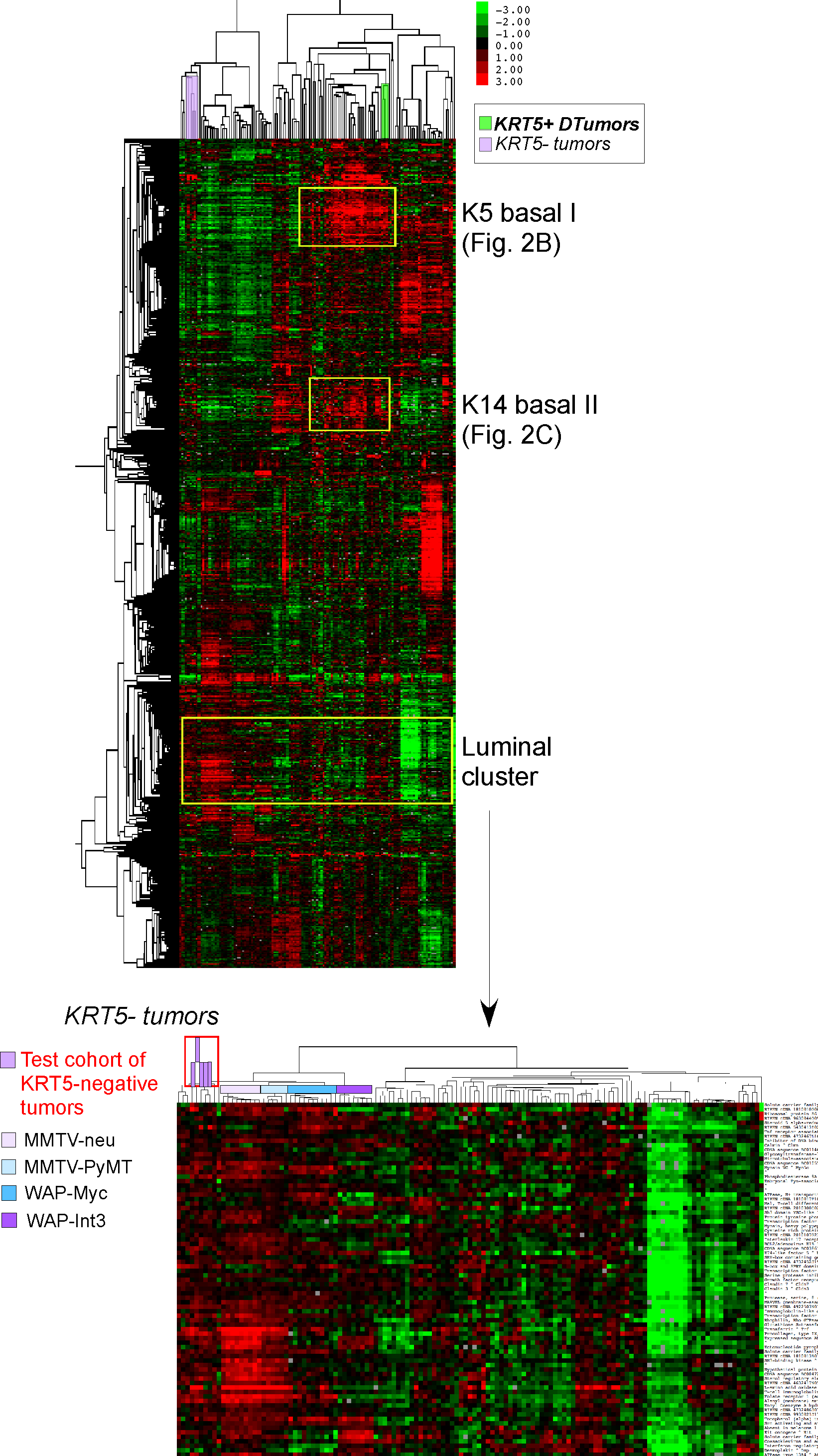

Supplement: Figure S2 — Comparison of the transcriptome of DTumors and KRT5-negative BALBc tumors with a bank of tumors from mouse models (previously described by Herschkowitz et al [30]). The two tumor types that arise in BALB/c mice in response to DMBA are shown on the overview of the heat-map, the basaloid group (in green) and the luminal group (in purple). Details of the KRT5-positive type I basal and KRT14-positive type II basaloid signatures are outlined (and illustrated in Fig. 2A–C). Samples designated as KRT5+ clustered together, but one (D19) was sufficiently plastic at the transcriptional level to express both basal and luminal genes. The luminal signature, boxed as (c), was highly represented in the KRT5-negative cohort (and excluded from the basaloid tumor type) and these tumors co-cluster with luminal tumors (including MMTV-expressing models such as MMTV-neu and MMTV-PyMT). One third-generation KRT5-negative tumor had a claudin-low signature. Detailed data and label expansions are available on request. (TIF) [file pone.0030979.s002.tif]

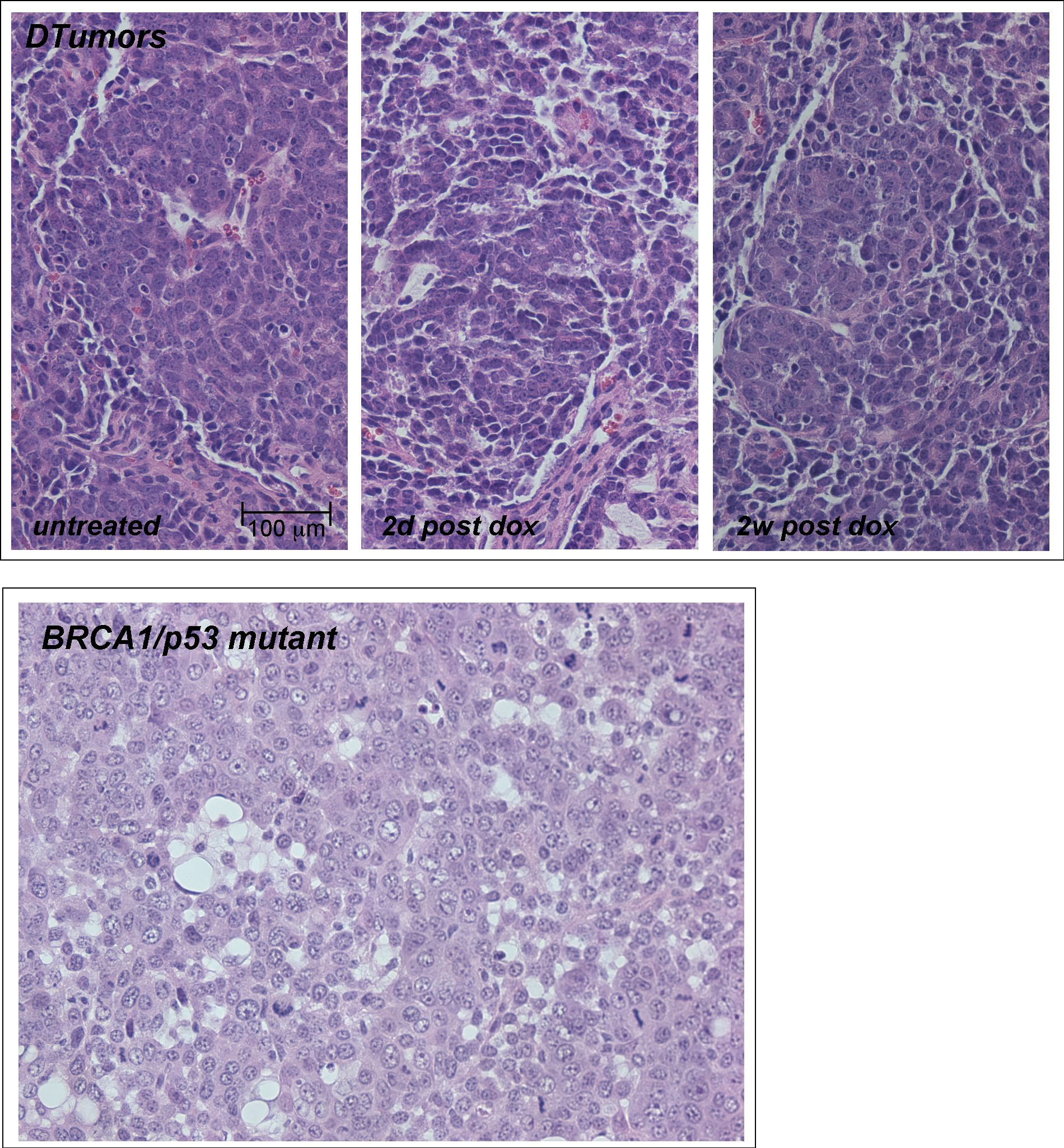

Supplement: Figure S3 — Histopathology of DTumors (treated with doxorubicin) and Brca1-p53 mutant mouse tumors. H&E stained paraffin sections of a representative DTumor, either untreated, or treated with doxorubicin for 2 days, or 2 weeks (as per Fig. 5) are shown, to illustrate their microacinar substructure, relatively lower stroma/interstitium with little evidence of inflammatory cells, lower proportion of necrotic areas/cells, low nuclear pleiomorphism and mid level mitotic index (typically grade II tumors). In contrast, a representative Brca1/p53 mutant tumor (illustrated by Molyneux et al [47]) shows higher rates of necrosis, high level nuclear polymorphism, substantial inflammatory infiltrate and high mitotic indices (not shown are their pushing margins). (TIF) [file pone.0030979.s003.tif]

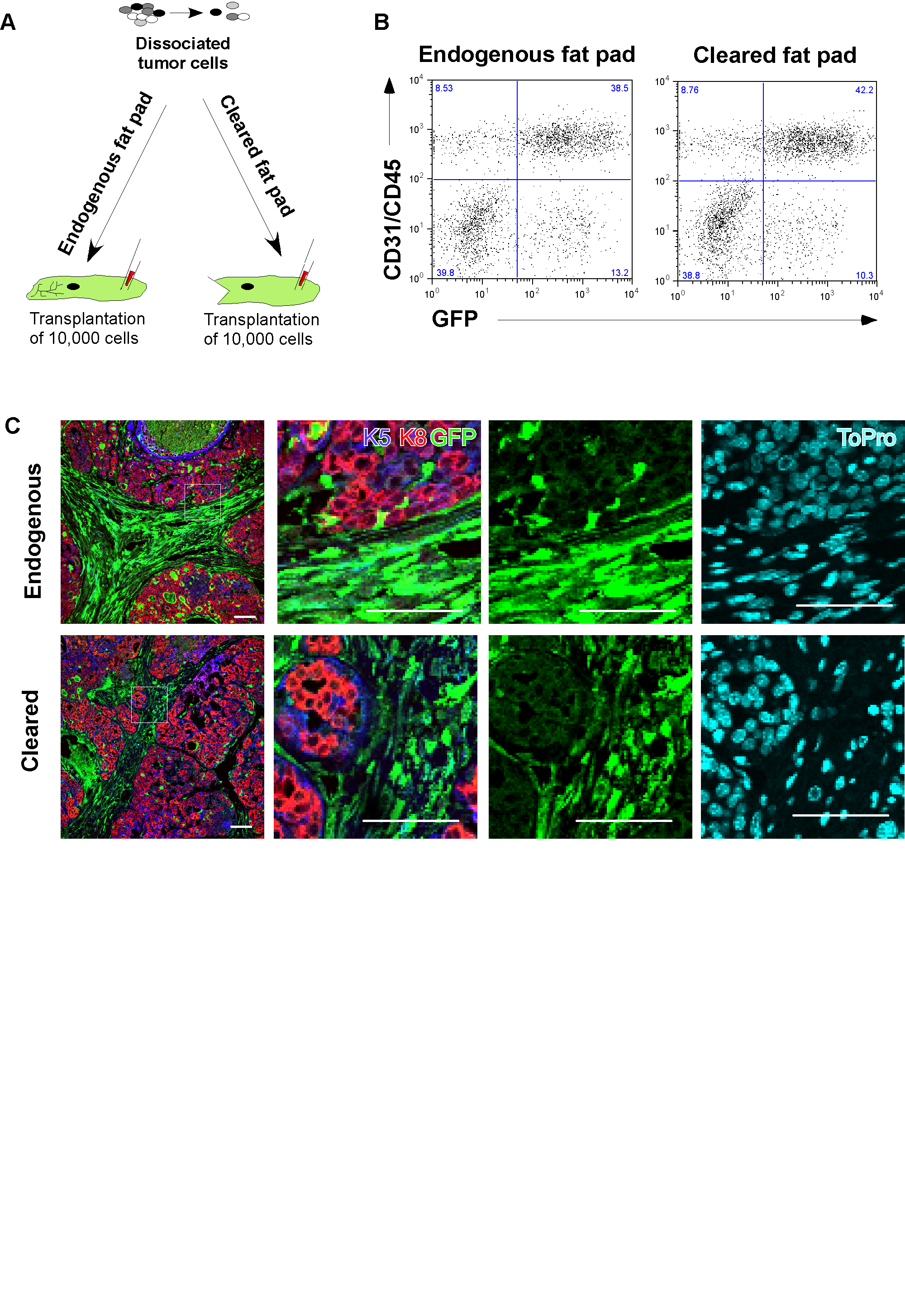

Supplement: Figure S4 — Only the stromal components are recruited from host, not either of the epithelial cell subtypes. (A) The experimental scheme to test for incorporation of cells from the host mammary gland into tumors. All cells in the host are GFP-positive, and isografted cells are unlabeled and transferred into either a cleared fat pad (no endogenous epithelium) or a normal mammary gland (with endogenous epithelium) to test whether endogenous mammary epithelial cells (say the basal cells) could be recruited to the growing tumor isograft, to generate the typical cell mixture. (B) Representative flow cytometric analysis of tumors growing in GFP-expressing recipients. Cells were stained for non-epithelial markers (so-called Lin+; CD31, (most) endothelial cells; CD45, (most) hematopoietic lineage), together with GFP (expressed by cells in the host fat pad). The quartiles represent cells that separate as follows: top right, non-epithelial tumor-associated cells derived from the host (Lin+ GFP+); bottom left, tumor epithelial cells derived from transplantation (Lin− GFP− cells). Epithelial cells recruited to the tumor from host are predicted to appear in the bottom right quartile (together with any non-epithelial cell types that turn out to be CD45−, CD31−). This assay illustrated the massive infiltration of non-epithelial GFP-positive cells to these tumors, and there was no difference between the fraction of CD31/CD45-negative cells with or without endogenous epithelium. (C) Visualization of GFP-positive cells in tumors. To confirm that GFP-positive cells were not epithelial, tumor sections were counterstained with luminal (K8, red) or basal (K5, blue) epithelial cell markers. Far left, low power; other panels are higher magnification (of area boxed); triple stained as indicated, or single stains to show cellular detail. Scale bar = 50 µm. (TIF) [file pone.0030979.s004.tif]

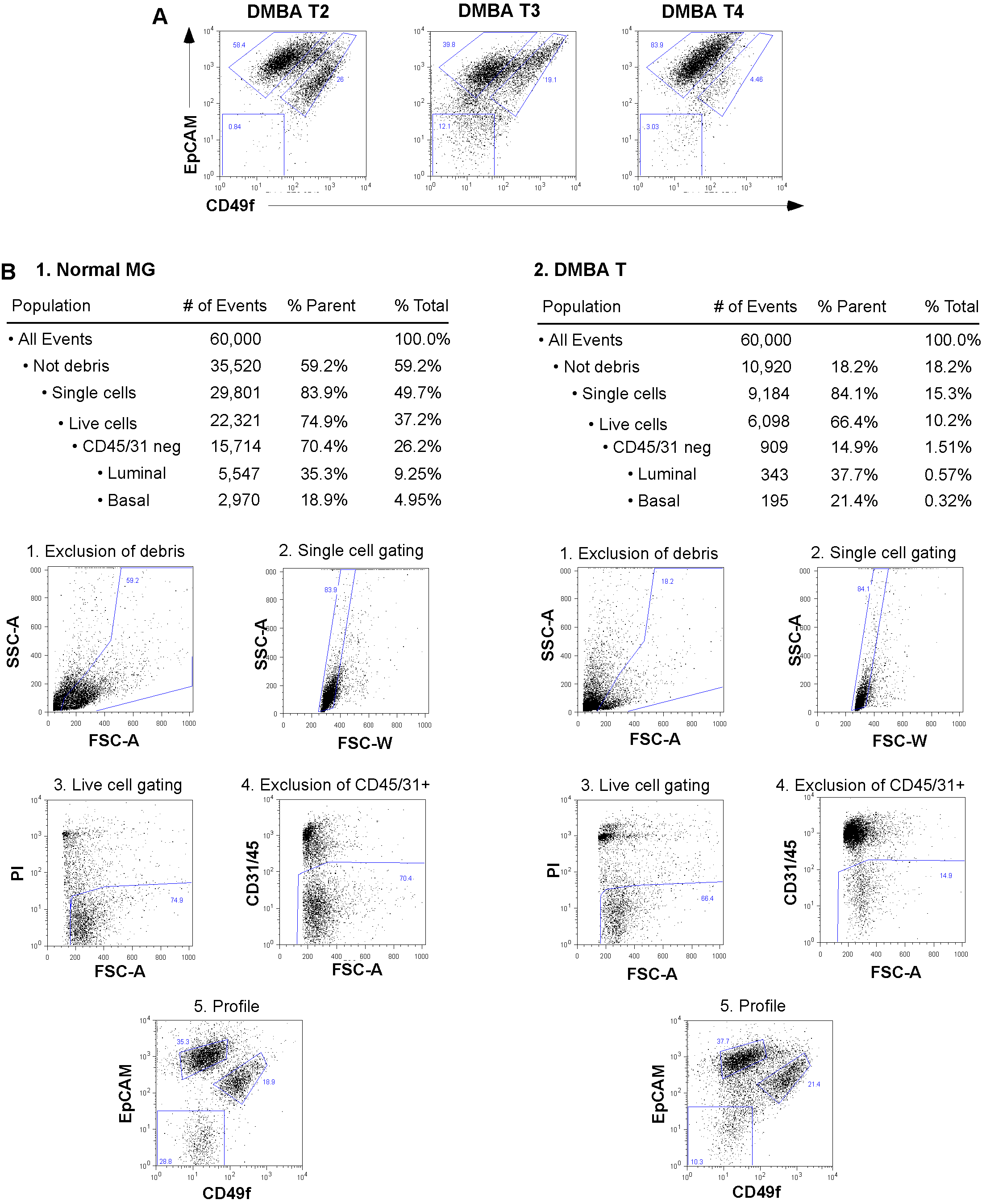

Supplement: Figure S5 — Gating and description of flow cytometric analysis and sorting. (A) More examples to display the typical separation of luminal and basal cells from several DTumors, based on their expression of EpCAM and CD49f. (B) The gating procedures used to separate luminal and basal epithelial cells from BALB/c mammary glands and DTumors. After gating out the debris and cell doublets, dead cells and lineage positive cells (Lin+) were sequentially excluded by staining with propidium iodide, and then CD45/CD31. The gating tree shows the proportion of events that were filtered through sequential gates. Single stained normal mammary gland epithelial cells were used as compensation controls and the automated compensation procedure were used with Diva Software. Cells were sorted with a 130 µm nozzle tip at low pressure (12 psi), and cells and 4-way sample collection tubes were maintained at 4°C. (TIF) [file pone.0030979.s005.tif]

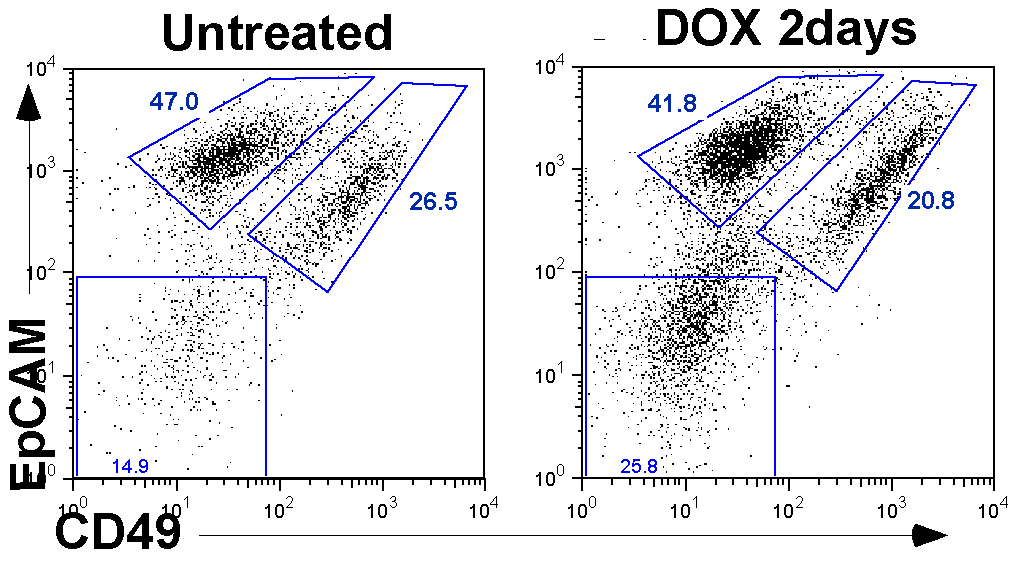

Supplement: Figure S6 — Flow cytometric analysis of untreated and doxorubicin-treated tumors. A representative cytogram of the cellular constituents of tumors (untreated and 2 days after administration of doxorubicin) shows that the proportion of each cell type is robustly maintained during treatment. (TIF) [file pone.0030979.s006.tif]

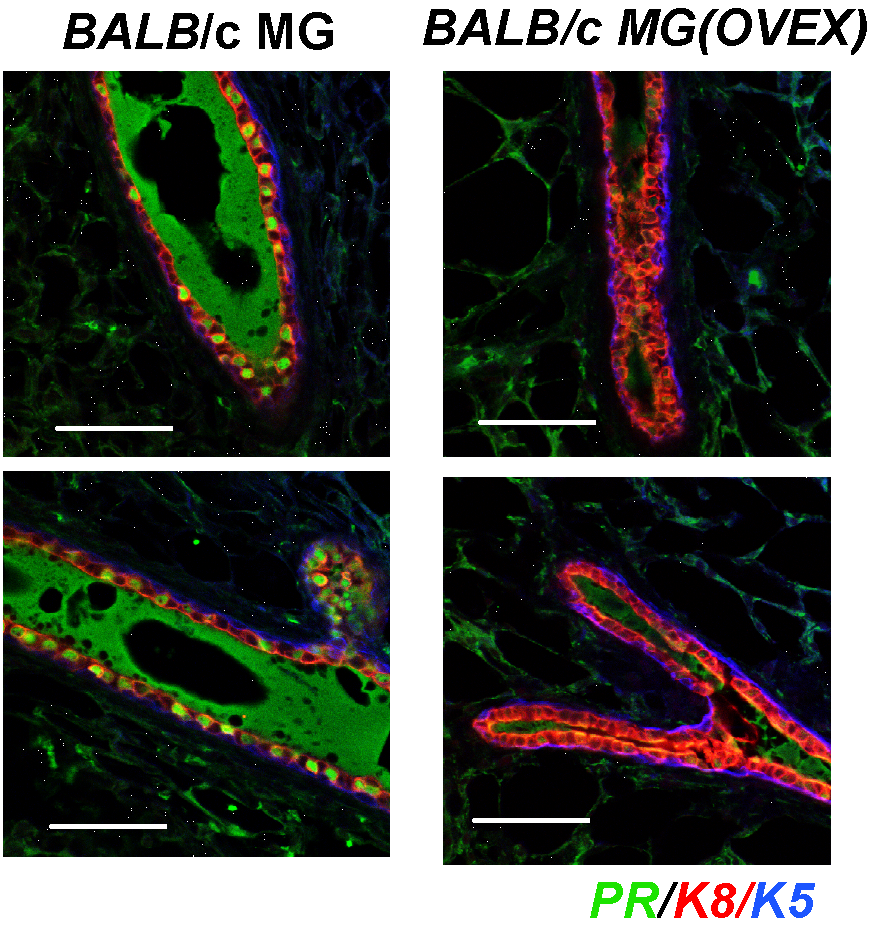

Supplement: Figure S7 — Demonstration of specificity of PRA staining. Immunohistochemical staining for PRA in normal virgin (MG) and ovarectomized BALB/c mammary glands, counterstained as indicated with both lineage markers, K5 (basal) or K8 (luminal). PRA staining is absent in ovarectomized glands, consistent with the loss of estrogen-ERα signaling. (TIF) [file pone.0030979.s007.tif]
